# *Tremella fuciformis* polysaccharides alleviate induced atopic dermatitis in mice by regulating immune response and gut microbiota

**DOI:** 10.3389/fphar.2022.944801

**Published:** 2022-08-25

**Authors:** Lingna Xie, Kaiye Yang, Yiheng Liang, Zhenyuan Zhu, Zhengqiang Yuan, Zhiyun Du

**Affiliations:** ^1^ School of Biomedical and Pharmaceutical Sciences, Guangdong University of Technology, Guangzhou, China; ^2^ Guangdong Provincial Key Laboratory of Plant Resources Biorefinery, Guangdong University of Technology, Guangzhou, China; ^3^ Infinitus Company Ltd., Guangzhou, China; ^4^ State Key Laboratory of Food Nutrition and Safety, Tianjin University of Science and Technology, Tianjin, China; ^5^ College of Food Science and Engineering, Tianjin University of Science and Technology, Tianjin, China

**Keywords:** T*remella fuciformis* polysaccharides, atopic dermatitis, immuno-modulatory, microbiota, metabolism

## Abstract

Atopic dermatitis (AD), characterized by severe pruritus, immune imbalance, and skin barrier dysfunction, has a high incidence worldwide. Recent evidence has shown that the modulation of gut microbiota is crucial for alleviating clinical symptoms of AD. Tremella fuciformis polysaccharides (TFPS) have been demonstrated to have a variety of biological activities such as immunomodulatory, anti-tumor, antioxidant, anti-inflammatory, neuroprotective, hypoglycemic and hypolipidemic effects. However, their effects on AD treatment have never been investigated. In this study, we compared the therapeutic effects of topical or oral administration of TFPS on AD in dinitrofluorobenzene (DNFB)-induced AD mice. Both topical application and oral administration of TFPS led to improvement on transdermal water loss, epidermal thickening, and ear edema in AD mice, but the oral administration showed significantly better efficacy than the topical application. The TFPS treatment increased the proportion of CD4 (+) CD25 (+) Foxp3 (+) regulatory T cells in mesenteric lymph nodes. Additionally, the non-targeted metabolomics and sequencing of 16S rDNA amplicons were performed, revealing metabolite modulation in feces and changed composition of gut microbiota in mice, which were induced for AD-like disorder and treated by oral administration of TFPS. Collectively, these data suggest that the oral administration of TFPS may constitute a novel effective therapy for AD, with underlying mechanisms associated with the regulation of immune response, and improvement of both metabolism and the composition of intestinal microbiota.

## Introduction

Atopic dermatitis (AD) is a chronic inflammatory skin disease, which is characterized by recurrent pruritus, dryness, eczematous skin lesions, and increasing incidence worldwide (Jin et al., 2020; Langan et al., 2020; Li et al., 2021). The pathogenesis of AD is associated with a variety of causes, such as an imbalance of Th1/Th2 responses (Stone et al., 1976), function defects of keratinocytes, elevated serum immunoglobulin E (Ig E), disturbed metabolism, and abnormal apoptosis of eosinophils. Topical application of corticosteroid creams is the common treatment for AD, and the systemic administration of immunosuppressant drugs or phototherapy (UV light) may be needed for reducing severe condition of the disease (Thomsen, 2014). However, these therapies cannot cure the disease, currently only aiming to alleviate its severity and duration. More effective therapeutic agents or treatment strategies are thus needed.

Normal skin can prevent antigens from entering the body and causing an inflammatory reaction because of the skin barrier function (Yang et al., 2020). However, skin barrier dysfunction in AD patients leads to enhanced penetration of allergens and transdermal infection of microorganisms, and damages to the stratum corneum of skin often leads to increased transepidermal water loss (TEWL) (Elias, 2005; Elias et al., 2008). Pro-inflammatory cytokines such as thymic stromal lipoprotein (TSLP), interleukin (IL)-25, and IL-33 can activate Th2 cells, which dominate early in the disease process and express cytokines such as IL-4 and IL-13 at high levels, leading to increase of IgE (Gandhi et al., 2016; Weidinger et al., 2018). In contrast, activation of Th1- and TH17-mediated responses have been reported in the development of chronic lesions in AD (Brunner et al., 2017).

The gut microbiota and its biochemical responses have an impact on many aspects of host health, including metabolism, immunity, development, and behavior (Sommer and Bäckhed, 2013; Belkaid and Hand, 2014; Collins, 2014; Behera et al., 2020). Gut microbes may play a role in the development of AD by regulating the immune system through interactions between the microbes and the host, and in particular, alterations in the gut microbiota affect immune system homeostasis by altering the production of metabolites. Several studies have demonstrated that the development of AD can be prevented by modulating the intestinal flora and metabolites (Fang et al., 2020; Kim et al., 2020; Chun et al., 2021).

*Tremella fuciformis* is a widely cultivated edible fungus in China. *T. fuciformis* polysaccharides (TFPS) has been identified as its main active ingredient. TFPS has a wide range of biological activities, including anticancer, antioxidant, anti-aging, and regulation on blood glucose and lipid. Recently, *T. fuciformis* polysaccharides (mainly 1 → 3-α-D-mannans) have been shown to alleviate dextran sodium sulfate (DSS)-induced colitis in mice through immunomodulation and restoration of intestinal microbiota and metabolites (Xu et al., 2021a). A recent study has revealed that TFPS was stable under simulated digestive conditions and could be utilized by the human fecal gut microbiota to increase short-chain fatty acid production (Wu et al., 2022). However, the TFPS potential for AD treatment has never been examined. It thus would be interesting to find out if TFPS can be used to alleviate AD or even cure the disease.

In this study, we assessed the therapeutic effect of TFPS on AD in a mouse AD model, and explored the possible action mechanisms by examining the effects of TFPS on Treg cells, gut microbiota, and gut microbes-derived metabolite composition in mice.

## Materials and methods

### Preparation of *Tremella fuciformis* polysaccharides

The TFPS used in this study was extracted from *T. fuciformis* by hot water extraction and ethanol precipitation. Briefly, *T. fuciformis* was ground into powder, mixed with 40 times of distilled water (W/W), and extracted in a 96°C water bath for 4 h. The supernatant was concentrated and precipitated with 75% ethanol. The crude polysaccharide extract was deproteinated by the Sevag method, and subsequently freezing-dried for further analysis.

### Composition analysis of *Tremella fuciformis* polysaccharides

The TFPS samples were hydrolyzed by trifluoroacetic acid to monosaccharide solution, and the composition was determined by using a Bruker Scion TQ triple quadruple mass spectrometer (Bruker, Fremont, California). Xylose, mannose, fucose, rhamnose, glucose, arabinose, and galactose were purchased (Sigma Aldrich, United States)and used as monosaccharide standards and identified according to the characteristic retention times.

### Establishment of dinitrofluorobenzene-induced allergic dermatitis in Babl/c mice

Six-week-old female Babl/c mice were purchased from the Experimental Animal Center in Sun Yat-sen University and fed libitum for 1 week before the experiment. After shaving hair on dorsal skin of mice, 0.25% (w/v) dinitrofluorobenzene (DNFB) in acetone and olive oil (3:1) was applied to both dorsal skin and ears of mice on day 1 and day4 at 100 μl/mouse and 25 μl/mouse, respectively. On day 7 and 10, animal dorsal and ear skins were further treated with 100 and 25 μl of 0.2% (w/v) DNFB, respectively. Balb/c mice were randomly divided into 8 experimental groups, including normal group (control), DNFB treatment group, 50 mg/kg prednidolone (PD) treatment group (including topical or oral administration of PD), 50 mg/kg TFPS treatment group (topical or oral administration of TFPS) and 200 mg/kg TFPS treatment group (topical or oral administration of TFPS). For the topical administration study, 100 μl of 12.5 mg/ml PD (50 mg/kg PD treatment group), 12.5 mg/ml TFPS (50 mg/kg TFPS treatment group) or 50 mg/ml TFPS (200 mg/kg TFPS treatment group) were applied to the animal dorsal skin, respectively. Neither normal group (control) nor the DNFB induction group was given any therapeutic treatments. Drug treatment was performed once a day and the epidermal recovery was examined. Treatment was carried out from day 5 to day 14. Animal feces were collected on day 15, and stored at −80°C; the dorsal skin was excised and stored at −80°C for further analysis.

### Evaluation of the severity of dermatitis in mice

The contact between the DNFB and the mouse ear skin stimulated itches making animals scratch. The intenser the itches, the more frequent scratching is observed. Skin symptoms were recorded on day 15. The severities of skin lesions were evaluated macroscopically as 4 levels: 0, no symptoms; 1, mild; 2, moderate; and 3, severe using the previously described scoring standards of atopic/eczema dermatitis syndrome concerning dryness/scaling, erythema/hemorrhage, and excoriation/erosion (Kang et al., 2017).

### Measurement of transepidermal water loss rates in dermatitis model mice

Transepidermal water loss (TEWL) rates on the dorsal skin of Babl/c mice were measured with the use of Multi Probe Adapter MCP 2 (CK, Germany), and the TEWL was recorded on day 5, day 8, day 11, and day 14, respectively.

### H&E staining

The skin tissue samples from different groups were immobilized in 4% formalin and embedded in paraffin. Each sample was deparaffined using xylene, rehydrated with a series of gradient alcohols, and stained with hematoxylin and eosin. Images were collected using a Nikon optical microscope (Japan) equipped with an eyepiece micrometer. All procedures are in accordance with the manufacturer’s guidelines.

### Immunohistochemistry

For immunohistochemical (IHC) staining, the fixed and paraffin-embedded skin samples were deparaffined, autoclaved, and heat-treated to recover antigens in citrate saline buffer. Tumor necrosis factor (TNF)-α and interferon (IFN)-γ were examined for expression in skin tissues by IHC. Stained sections were examined and imaged by using a Nikon optical microscope (Japan) equipped with an eyepiece micrometer under the ×200 magnification.

### Enzyme-linked immunosorbent assay

The concentration of IgE in serum was determined by the ELISA assay. A mouse IgE ELISA assay kit was purchased (Jiangsu Meibiao Biological Technology Co. Ltd., Jiangsu, China) and used to detect the serum IgE in each test group following the manufacturer’s instructions.

### Flow cytometry analysis

Mesenteric lymph nodes were isolated from each group of mice, and lymphocytes were isolated by a mouse lymphocyte isolation kit. Lymphocyte suspensions were prepared and adjusted to 2.0 × 10^6^ cells per vial in RPMI 1640 medium containing 10% fetal bovine serum (FBS). Then prepared cells were labelled for 20 min in ice bath using the fluorescein isothiocyanate (FITC)-conjugated anti-mouse CD4 antibody (ab), phycoerythrin (PE)-conjugated anti-mouse CD25 ab, and allophycocyanin (APC)-conjugated anti-mouse Fork head box (Fox) P3 ab. The labeled samples were analyzed by flow cytometry (FlowSight, Merck Millipore), and data was analyzed using FlowJo software (Tree Star Inc. Ashland, OR, United States).

### 16S rDNA amplicon sequencing

Microbial community genomic DNA was extracted from feces samples using the E. Z.N.A.^®^ soil DNA Kit (Omega Bio-Tek, Norcross, GA, U.S.) according to the manufacturer’s instructions. The DNA extract was checked on 1% agarose gel, and DNA concentration and purity were determined with NanoDrop 2000 UV-vis spectrophotometer (Thermo Scientific, Wilmington, United States). The hypervariable region V3-V4 of the bacterial 16S rRNA gene was amplified with primer pairs 338F (5′-ACT​CCT​ACG​GGA​GGC​AGC​AG-3′) and 806R (5′-GGACTACHVGGGTWTCTAAT-3′) by an ABI GeneAmp^®^ 9700 PCR thermocycler (ABI, CA, United States). The PCR amplification of the 16S rRNA gene was performed as follows: initial denaturation at 95°C for 3 min, followed by 27 cycles of denaturing at 95°C for 30 s, annealing at 55°C for 30 s, and extension at 72 °Cfor 45 s, and single extension at 72°C for 10 min, and end at 4°C. The PCR mixtures contain 5 × TransStart FastPfu buffer 4 μl, 2.5 mM dNTPs 2 μl, forward primer (5 μM) 0.8 μl, reverse primer (5 μM) 0.8 μl, TransStart FastPfu DNA Polymerase 0.4 μl, template DNA 10 ng, and finally ddH2O up to 20 μl. PCR reactions were performed in triplicate. The PCR product was extracted from 2% agarose gel and purified using the AxyPrep DNA Gel Extraction Kit (Axygen Biosciences, Union City, CA, United States) according to the manufacturer’s instructions and quantified using Quantus™ Fluorometer (Promega, United States).

Purified amplicons were pooled in equimolar and paired-end sequenced on an Illumina MiSeq PE300 platform/NovaSeq PE250 platform (Illumina, San Diego, United States) according to the standard protocols by Majorbio Bio-Pharm Technology Co. Ltd. (Shanghai, China). The raw reads were deposited into the NCBI Sequence Read Archive (SRA) database.

The raw 16S rRNA gene sequencing reads were demultiplexed, quality-filtered by fastp version 0.20.0 (Chen et al., 2018), and merged by FLASH version 1.2.7 (Chen et al., 2018) with the following criteria: 1) the 300 bp reads were truncated at any site receiving an average quality score of <20 over a 50 bp sliding window, and the truncated reads shorter than 50 bp were discarded, reads containing ambiguous characters were also discarded; 2) only overlapping sequences longer than 10 bp were assembled according to their overlapped sequence. The maximum mismatch ratio of the overlap region is 0.2. Reads that could not be assembled were discarded; 3) Samples were distinguished according to the barcode and primers, and the sequence direction was adjusted, exact barcode matching, 2 nucleotide mismatches in primer matching.

Operational taxonomic units (OTUs) with a 97% similarity cutoff (Stackebrandt and GOEBEL, 1994; Edgar, 2013)were clustered using UPARSE version 7.1, and chimeric sequences were identified and removed. The taxonomy of each OTU representative sequence was analyzed by RDP Classifier version 2.2 (Wang et al., 2007) against the 16S rRNA database (eg., Silva v138) using a confidence threshold of 0.7.

### Metabolomics profiling of feces samples

Before analysis, the feces samples were thawed at 4°C, diluted (80 mg of feces in 200 μl of purified water), vortex-shocked for 20 s, and then centrifuged at 13,000 g for 10 min at 4°C, and the resulting supernatant was transferred to an EP tube. To the precipitate obtained by centrifugation, 200 μl of methanol was added, shaken and mixed, and centrifuged at 13,000 g for 10 min at 4°C, and the resulting supernatant was again added to the EP tube. To the precipitate obtained by centrifugation, 200 μl of acetonitrile was added, shaken, and mixed, and centrifuged at 13,000 g for 10 min at 4°C. The supernatant was again added to the EP tube described above. The mixture in the EP tube was shaken well, centrifuged at 13,000 g for 10 min at 4°C, and passed through a 0.22 microporous membrane in preparation for the HRAM LC-MS/MS analysis. A 20 μl volume from each sample supernatant was mixed to prepare quality control samples.

A Thermo Scientific Ultimate 3000 RSLC and a Q Exactive Orbitrap desktop high-resolution mass spectrometer was used to carry out the HRAM LC-MS/MS analysis. Thermo Scientific Xcalibur was used for data collection, and Compound Discoverer 2.1, mzCloud database (Thermo Scientific, HTTP://www.mzcloud.org) was used for data analysis. The data was analyzed using Compound Discoverer 2.1 to measure the statistical differences of metabolites between each group. MetaboAnalyst (https://www.metaboanalyst.ca/) was used to enrich the metabolic pathways of significantly different metabolites.

### Analysis of short-chain fatty acids

Two hundred mg of mice fecal sample was extracted with 5 ml of 70% isopropanol and then subjected to 3-NPH derivatization. Standard short-chain fatty acid (SCFA) solutions (acetic acid, propionic acid, butyric acid, isobutyric acid, valeric acid, isovaleric acid, and hexanoic acid) were run at 6 different concentrations. The data were analyzed using Multiquant (SCIEX). Concentrations of SCFA were normalized to the weight of fecal sample (mg).

A Thermo Scientific Ultimate 3000 RSLC that is coupled to a 4000 QTRAP triple quadrupole mass spectrometer (AB Sciex, Concord, ON, Canada) equipped with an ESI source and operating in negative ion mode was used. Chromatographic separations were performed on a BEH C_18_ (2.1 × 100 mm, 1.7 μm, Waters) UPLC column using water: formic acid (100:0.01, v/v; solvent A) and methanol: formic acid (100:0.01, v/v; solvent B) as the mobile phases for gradient elution. The column flow rate was 0.35 ml/min; the column temperature was set at 50°C and the autosampler was kept at 5°C.

### Statistical analysis

Quantitation data were presented as means ± SD, and differences between the three groups were analyzed using one-way analysis of variance (ANOVA) and Tukey’s post hoc test. A correlation analysis was used to analyze the correlation between changes of intestinal flora composition and metabolite levels, and the Pearson correlation coefficient was selected. A *p*-value < 0.05 was considered statistically significant.

## Results

### Preparation and characterization of *Tremella fuciformis *polysaccharides

**FIGURE 1:**
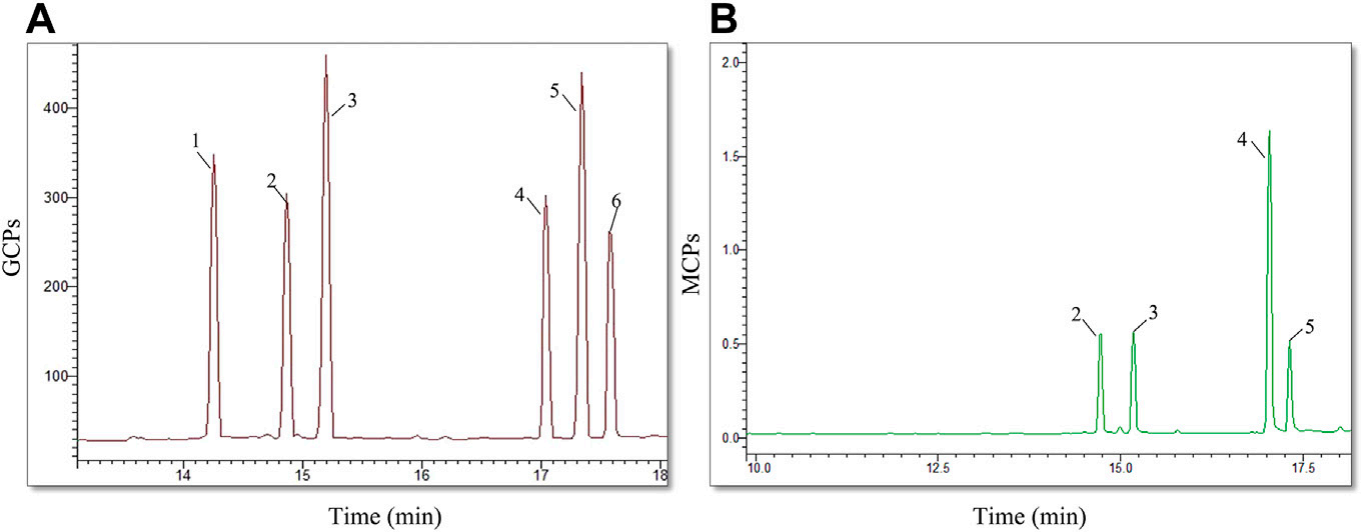
The monosaccharide composition analysis. **(A)**The chromatogram of mixed monosaccharide standards, showing a composition of rhamnose (1), arabia sugar (2), xylose (3), mannose (4), glucose (5), galactose (6). **(B)** The chromatogram of TFPS hydrolysate shows the monosaccharide composition of arabia sugar (2), xylose (3), mannose (4), and glucose (5).

The Tremella fuciformis mushroom was ground and extracted for polysaccharide preparation in hot distilled water. After deproteinization, the extracted TFPS fraction was measured by high-performance gel permeation chromatography (HPGPC), revealing molecular weights ranging from 30 to 1,230 kDa. This indicates that the prepared product is not a single polysaccharide, but a mixture of different polysaccharides. Subsequently, the extracted TFPS was hydrolyzed in trifluoroacetic acid and analyzed for monosaccharide composition by mass spectrometry. As shown in Figure 1, the prepared TFPS is composed of xylose, mannose, arabia sugar and glucose at a molar ratio of 1.3:3.4:1.0:1.0.

### *Tremella fuciformis* polysaccharides is effective for attenuating the development of dinitrofluorobenzene-induced Atopic dermatitis-like symptoms in mice

**FIGURE 2:**
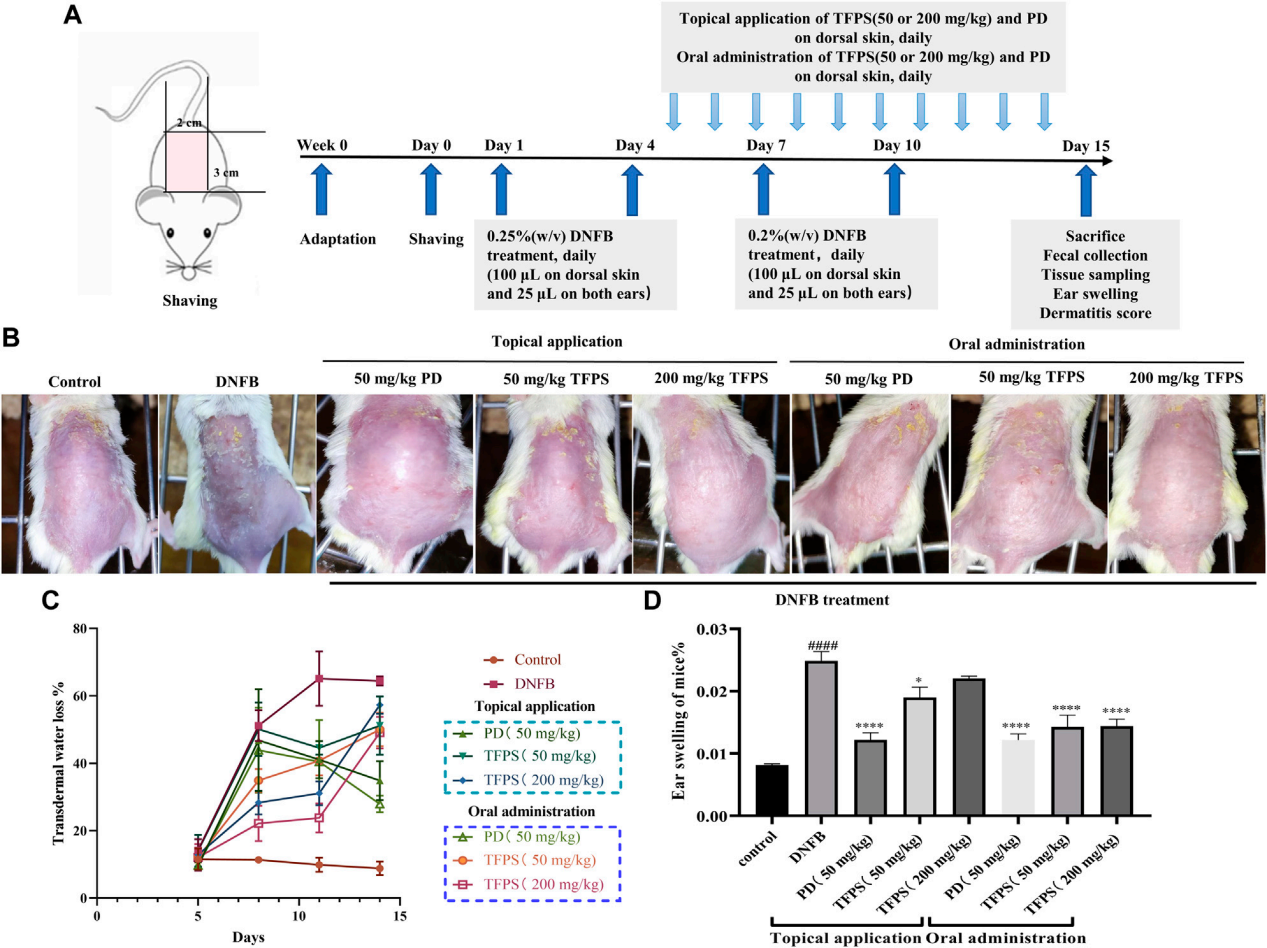
Improvement of AD-like symptoms by TFPS treatment in a murine AD model. **(A)** *In vivo* experimental schedule is shown. Babl/c mice were divided into eight groups: normal control (Control group), DNFB + PBS (DNFB group), 50 mg/kg PD group (including topical or oral administration of PD), 50 mg/kg TFPS treatment group (including topical or oral administration of TFPS) and 200 mg/kg TFPS treatment group (including topical or oral administration of TFPS). To study the effect of TFPS on atopic dermatitis, mice were treated with DNFB on day 1, day 4, day 7, and day 10, then topical or oral administration of TFPS for 10 days after the DNFB treatment. **(B)** The mouse taken on the 14th day is rephotographed. **(C)**The TEWL was tested by electrolyzed water analysis on day 5, day 8, day 11, and day 14. **(D)** Animals were culled and animal ears were removed for punch weighing on day 15. All values are presented as means ± S.E.M (n = 3). ###*p* < 0.001, compared with Control group. ∗*p* < 0.05, ∗∗∗*p* < 0.0001, compared with DNFB group.

The therapeutic efficacy of TFPS was evaluated in a DNFB-induced murine AD model. As scheduled in Figure 2A, DNFB was applied on mice dorsal and ear skins to induce AD-like symptom. As shown in Figure 2B, DNFB successfully stimulated AD-like symptoms, including erythema/bleeding, edema, shedding/erosive, and dry/desquamation. One previous study had used 40 mg/kg of prednisolone for the treatment of contact dermatitis, which showed effective and no significant toxic side effects (Takeuchi et al., 2010). Thus, a similar dose of prednisolone (50 mg/kg) was tested in this study. Previously, Xu et al. have demonstrated the good tolerance of oral administration of 200–400 mg/kg TFPS (Xu et al., 2021a), thus 50 mg/kg and 200 mg/kg of TFPS treatment were tested for AD treatment, respectively, in this study.

Of note, both TFPS and positive drug (PD) prednidolone treatments attenuated the development of AD condition on mice. The measurement of transepidermal water loss (TEWL) was performed in dorsal skin of mice to assess therapeutic efficacies. The obtained results showed that the DNFB stimulation caused significant increase of TEWL (Figure 2C). However, both topical application and oral administration of TFPS successfully attenuated the TEWL of dorsal skin. Moreover, mice were sacrificed on day 15, and animal ears were removed, punched and weighed to examine the effect of TFPS treatment on reducing the severity of ear swelling induced by DNFB (Figure 2D). The significant increase of ear weight in DNFB group suggested that the severe ear swelling was induced in mice by the DNFB irritation. Interestingly, both topical application and oral administration of TFPS were effective for reducing the ear swelling. However, the oral administration of TFPS appeared more effective than topical application. Collectively, these data demonstrated that TFPS treatment was effective for improving the DNFB-induced AD-like symptoms in mice.

### *Tremella fuciformis* polysaccharides treatment suppressed dinitrofluorobenzene-induced epidermal thickening and mast cell infiltration in mice

**FIGURE 3:**
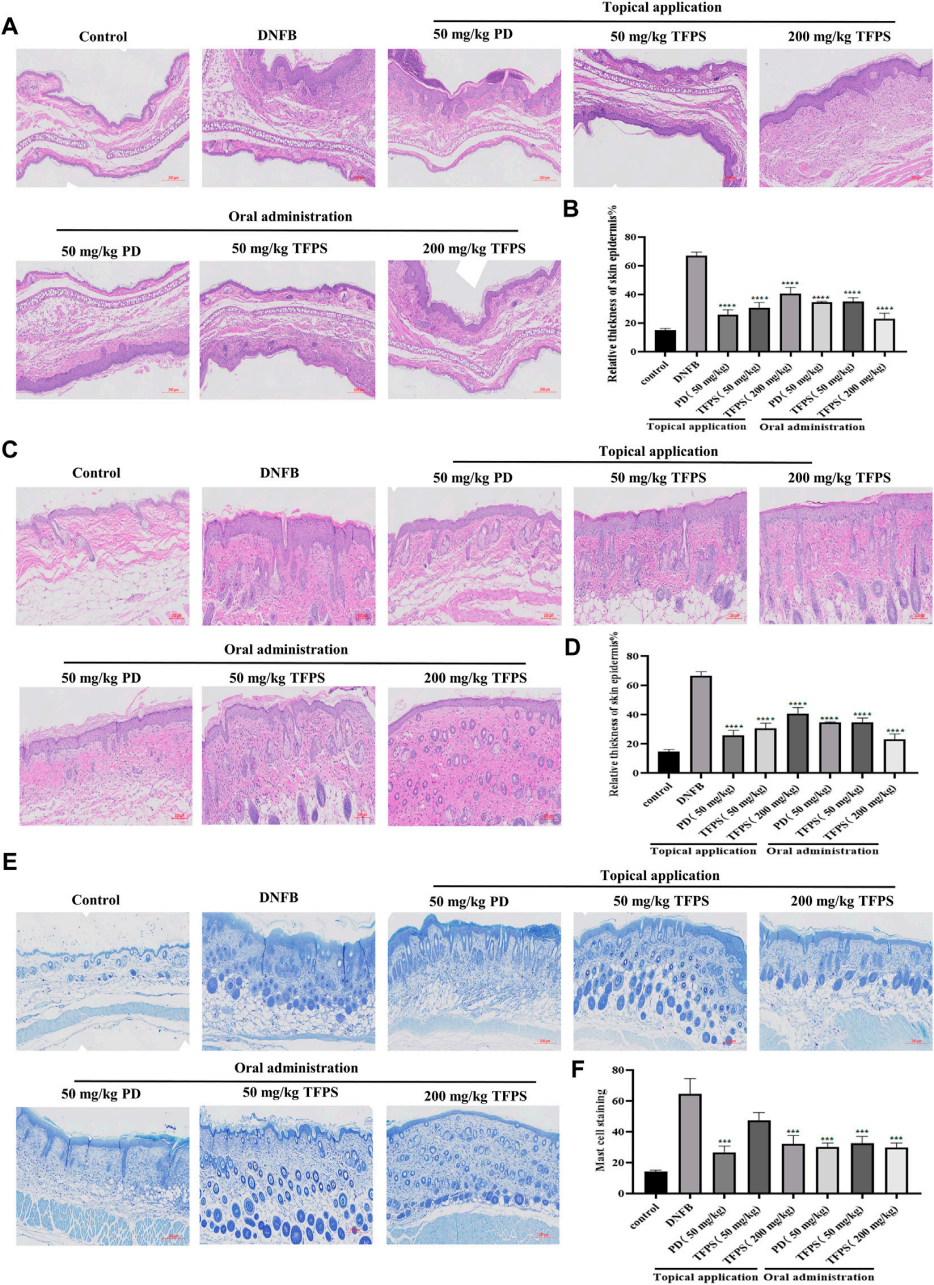
Effects of TFPS on the histomorphological changes in mice with DNFB induced AD-like symptoms. **(A)** HE stained section of ear tissue (magnification ×300) **(B)** The measurement of ear skin epidermal thickness in different experimental groups is presented as relative to that of control group, for which the value is set as 100%. **(C)** HE staining of dorsal skin tissues (magnification ×400) **(D)** The measurement of dorsal skin epidermal thickness in experimental groups is presented as relative to that of the control group, for which the value is set as 100%. **(E)** Staining of mast cells in the dorsal skin tissue (magnification ×300) **(F)** Calculate the number of mast cells at random locations. All values are represented as means ± S.E.M (*n* = 3). ###*p* < 0.001, compared with the control group. ∗*p* < 0.05, ∗∗*p* < 0.01, ∗∗∗*p* < 0.001, compared with the DNFB group.

Next the effects of TFPS on skin barrier function were examined. The H&E staining of animal ear and dorsal skins revealed evident epidermal hyperkeratosis, thickened stratum corneum, and severe AD-like lesions induced by DNFB irritation in mice (Figures 3A,C). Notably, both topical application and oral administration of TFPS significantly reduced the DNFB-induced epidermal thickening, with comparable effectiveness with the positive control drug PD (Figures 3A–D). Importantly, the oral administration of TFPS appeared better reduction of epidermal thickening than the topical application of TFPS at the dose of 200 mg/kg. Additionally, mast cell infiltration was investigated by toluidine blue staining, revealing that the DNFB irritation induced significant mast cell infiltration compared with the normal control (Figure 3E). Interestingly, like the PD drug, the TFPS treatment by either topical application or oral administration showed significant suppression on the mast cell infiltration (Figures 3E,F). These observations indicate that TFPS can improve the skin barrier function and thus is beneficial for AD treatment.

### *Tremella fuciformis* polysaccharides treatment reduced the inflammatory response in dinitrofluorobenzene-induced mice

**FIGURE 4:**
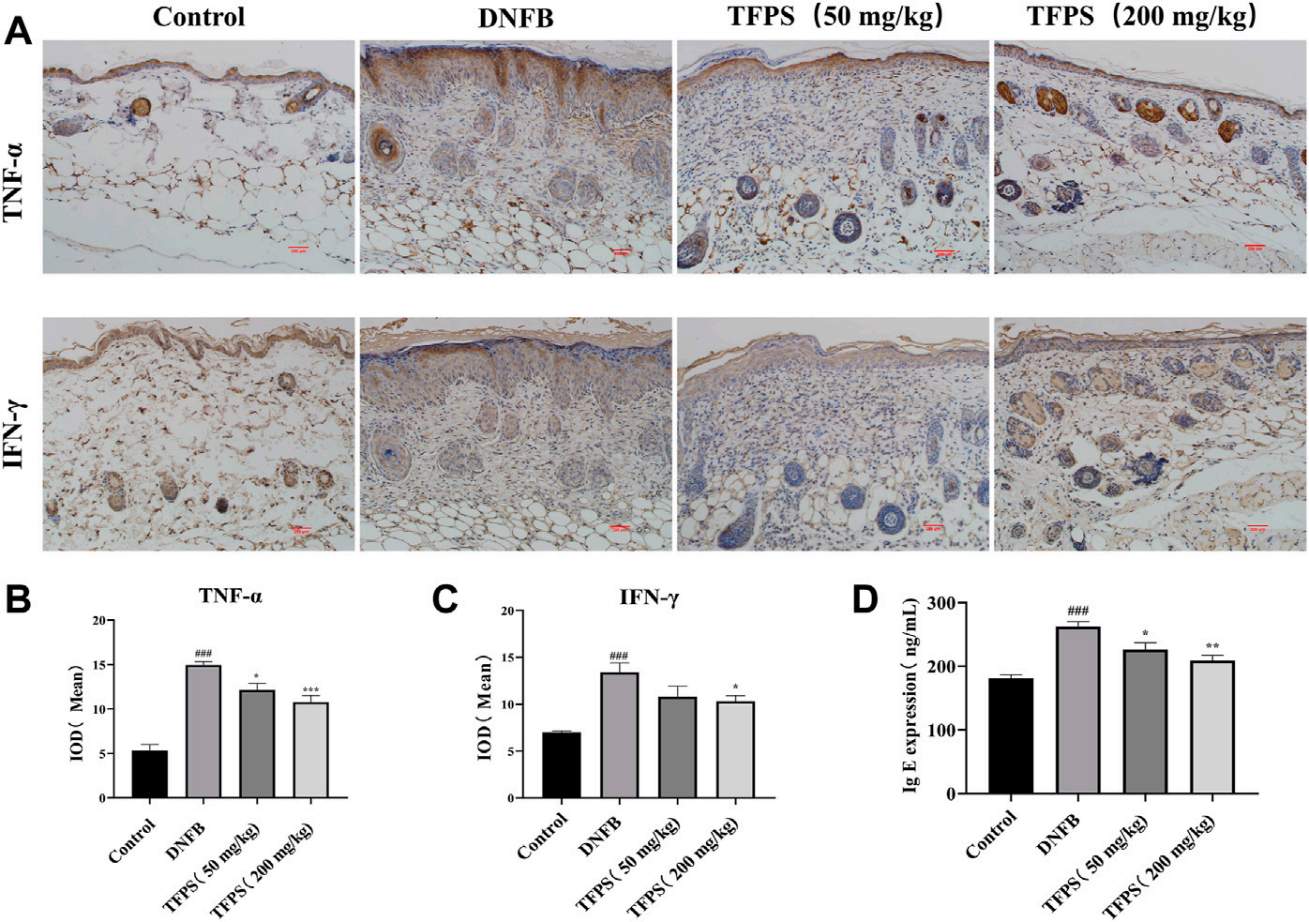
Effect of oral administration of TFPS on cytokine expression in the dorsal skin of AD mice. **(A)** The IHC results of TNF-α and IFN-γ in kidney of Control, DNFB and TFPS(50 or 200 mg/kg) groups (magnification ×200) **(B)** Mean IOD of TNF-α group. **(C)** Mean IOD of IFN-γ IHC in each group. **(D)** Determination of serum IgE level in each test group. All values are represented as means ± S.E.M (*n* = 3). ###*p* < 0.001, compared with Control group. ∗*p* < 0.05, ∗∗*p* < 0.01, ∗∗∗*p* < 0.001, compared with DNFB group.

To detect cytokine levels in dorsal skin, we performed immunohistochemistry assessment on TNF-α and IFN-γ (Figure 4A), respectively, and the mean integral optical density (IOD) of TNF-α and IFN-γ IHC were shown in Figures 4B,C. The obtained results showed that TFPS treatment significantly reduced TNF-α and IFN-γ expression in DNFB-induced AD mice. In addition, animal serum IgE expression was found to be significantly upregulated in AD mice by ELISA comparing with that in control group (Figure 4D). However, the IgE level was significantly reduced after TFPS treatment in the AD mice, indicating that the TFPS treatment could alleviate DNFB-induced inflammatory responses.

### *Tremella fuciformis* polysaccharides treatment increases the treg cell population in the mesenteric lymph nodes

The CD4 (+) CD25 (+) FOXP3 (+) regulatory T (Treg) cells stem from the further differentiation of CD4 (+) T cells, and play essential roles in the regulation of excessive immunity and maintenance of immune tolerance. The pathogenesis of AD is not fully understood yet and it is not known if Treg cells participate in the immune modulation of AD. To address this issue, lymphocytes were isolated from animal mesenteric lymph nodes, immuno labelled and analyzed for Treg ratio to CD4 (+) T lymphocytes by flowcytometry.

**FIGURE 5:**
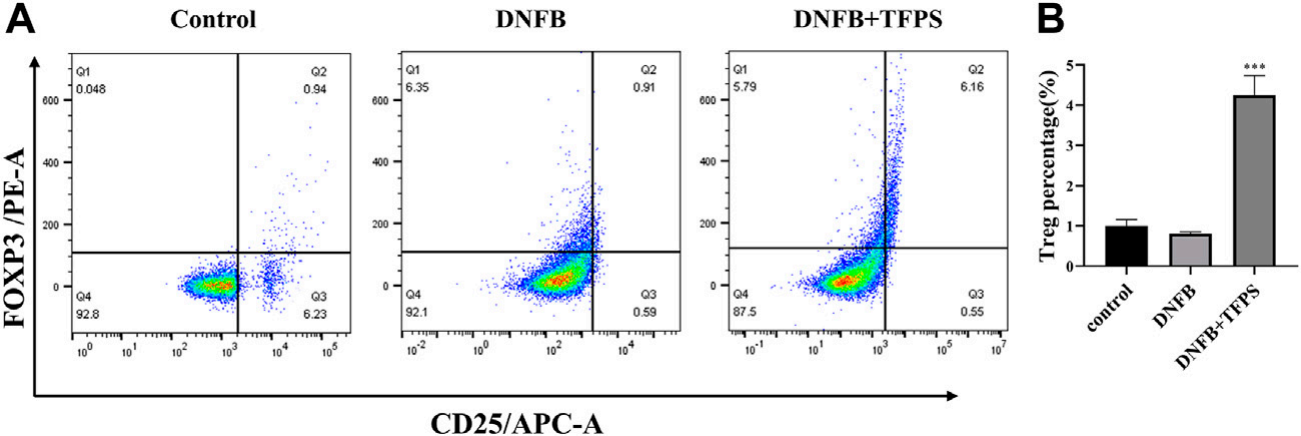
Effects of oral administration of TFPS on Treg number in mesenteric lymph nodes (MLNs) of AD mice. **(A)** The MLN cells were isolated from babl/c mice treated by vehicle control, DNFB, or DNFB plus 200 mg/kg TFPS. **(B)** The number of CD4+ CD25+ Foxp3+ cells was calculated based on the percentage of total MLN cell counts. All values are represented as means ± S.E.M (*n* = 3). ∗∗∗*p* < 0.001, compared with the DNFB group.

As shown in Figures 5A,B, the DNFB stimulation slightly downregulated the Treg ratio in mice compared with normal control. However, oral administration of TFPS (200 mg/kg) significantly upregulated the Treg ratio in DNFB-induced AD mice. This observation suggests that TFPS may attenuate AD development via increasing the number of Treg cells to modulate excessive immune responses.

### The influence of *Tremella fuciformis* polysaccharides on the taxonomic composition of the gut microbiota

**FIGURE 6:**
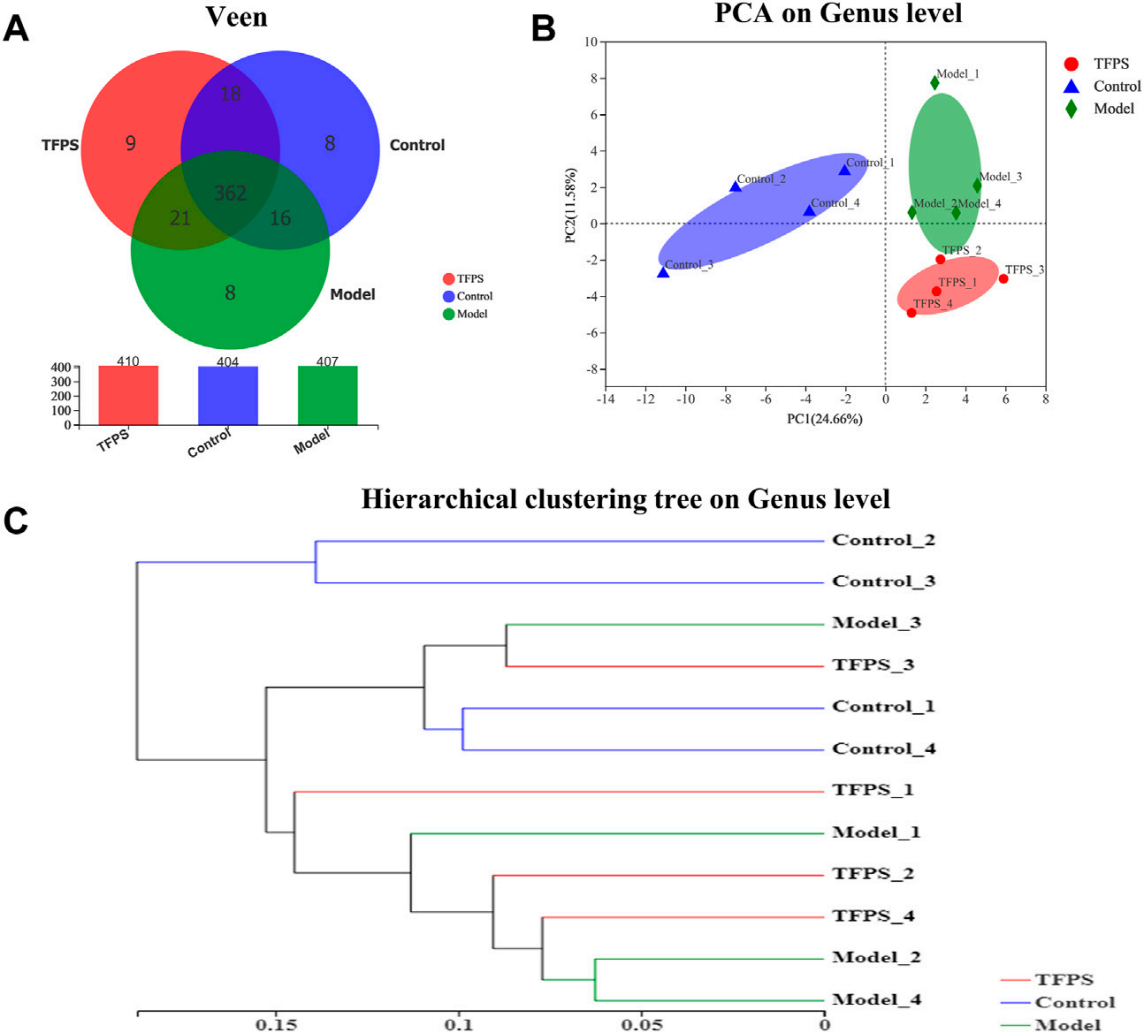
TFPS relieves DNFB-induced microbiota changes in AD-like mice. **(A)**Venn diagrams of OTUs. **(B)** PCA plot with unweighted UniFrac. **(C)** UPGMA clustering results based on unweighted UniFrac distance of microbial 16S rRNA sequences from animal feces samples.

The dynamic interaction of the gut microbiota with other microorganisms and the host is vital to the health of the host. We conducted 16s rRNA sequencing analysis on mouse feces to study the effect of TFPS on the composition of the intestinal microbiota in DNFB-induced AD mice. We obtained a total of 495,630 high-quality 16s rRNA reads. After diluting the samples to the same sequencing depth, the minimum sequence number is 26,008. In three groups of samples, the bacteria belonged to 9 phyla, 14 classes, 31 orders, 48 families, 107 genera, 175 species and 442 OTUs were identified. A Venn diagram was constructed to examine the existence of OTUs with a relative abundance >0.1% in each group (Figure 6A). Most OTUs (362 in all) were shared by all three groups. However, a total of 18 OTUs were specifically shared by the control and the TFPS treatment groups. Additionally, a total of 21 OTUs were shared by only the control and the model groups, and 16 OTUs were shared by only the model and the TFPS treatment groups. In total, the unique OUTs of the control group, model group, and TFPS group were 8, 8, and 9, respectively.

The intestinal microbiota diversity of the control group, the model group, and the TFPS group is shown in Table 1. We observed no significant difference in the chao1 index or the Simpson index among the three groups. In order to evaluate the changes in intestinal flora *ß* diversity between different groups, principal coordinate analysis based on unweighted uniform distance matrix analysis was performed (Figure 6B). The PCA results showed a significant separation of bacterial composition in the control group, model group, and TFPS group, and there were significant differences in bacterial communities in the different groups. These findings suggested that TFPS can improve the intestinal flora composition of AD-like mice.

**TABLE 1:** Effects of TFPS on the *a*-diversity index of gut microbiota.

	Control	Model	TFPS
Shannon	2.872 ± 0.090	2.602 ± 0.018	2.533 ± 0.053
Simpson	0.105 ± 0.011	0.130 ± 0.004	0.166 ± 0.017
Ace	91.513 ± 3.081	92.001 ± 1.273	89.299 ± 0.765
Chao 1	88.900 ± 2.456	89.433 ± 1.402	87.621 ± 1.218

The UPGMA cluster analysis was performed using the unweighted UniFrac method, and the obtained results were shown as a dendrogram (Figure 6C), which revealed that the species composition of animal gut microbiota was significantly changed in DNFB-induced AD mice, however the TFPS treatment can revert the changes.

**FIGURE 7:**
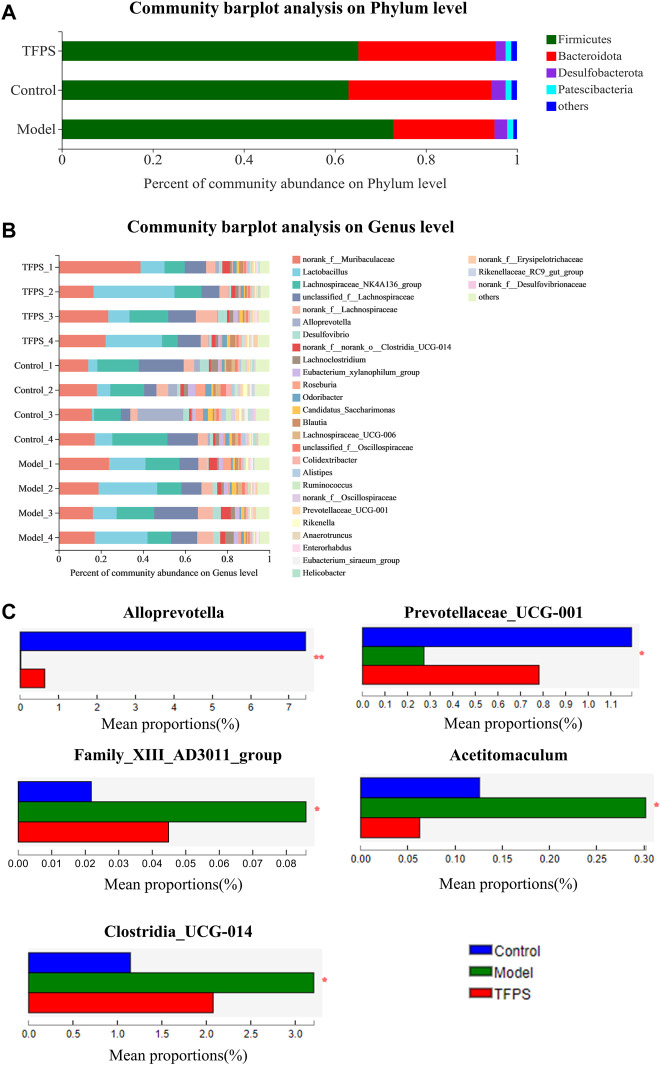
**(A)**Effects of TFPS on the relative abundance of gut microbiota at the phylum level. **(B)** Relative abundance of gut microbiota at the genus level. **(C)** Comparison of the relative abundance of significantly different microbial groups at the genus level. The Mann-Whitney *U* test was used for statistical analysis, and the false discovery rate (FDR)-corrected, compared with Model group (*N* = 4 mice per group). ∗, *p* < 0.05, ∗∗, *p* < 0.01.

According to taxonomic analysis, the relative abundance of each phylum was calculated, revealing that *Firmicutes*, *Bacteroides*, *Desulfobacteria*, *Proteobacteria*, and *Actinomycetes* are the mainly detected bacterial phyla in the feces microbiota (Figure 7A). Our study found that Firmicutes and Bacteroidetes accounted for more than 90.0% of the total gut microbial composition in all analyzed samples. The phyla F/B ratio of the gut microbiota was higher in DNFB-induced mice (3.72 ± 0.18) than in the control group (1.72 ± 0.98). Notably, the oral administration of TFPS stimulated the expansion of Bacteroidetes populations but reduced the Firmicute populations, leading to the restoration of F/B ratio to 1.83 ± 0.50. Taxonomic-based analysis suggested that Alloprevotella and Prevotellaceae-UCG-001 increased significantly after TFPS treatment, while Acetitomaculum, Family XⅢ AD3011_group and Clostridia_UCG-014 decreased significantly in mouse gut microbiota, indicating that the composition of gut microbiota was significantly modulated by TFPS treatment in mice (Figures 7B,C).

### Metabolomics analysis

**FIGURE 8:**
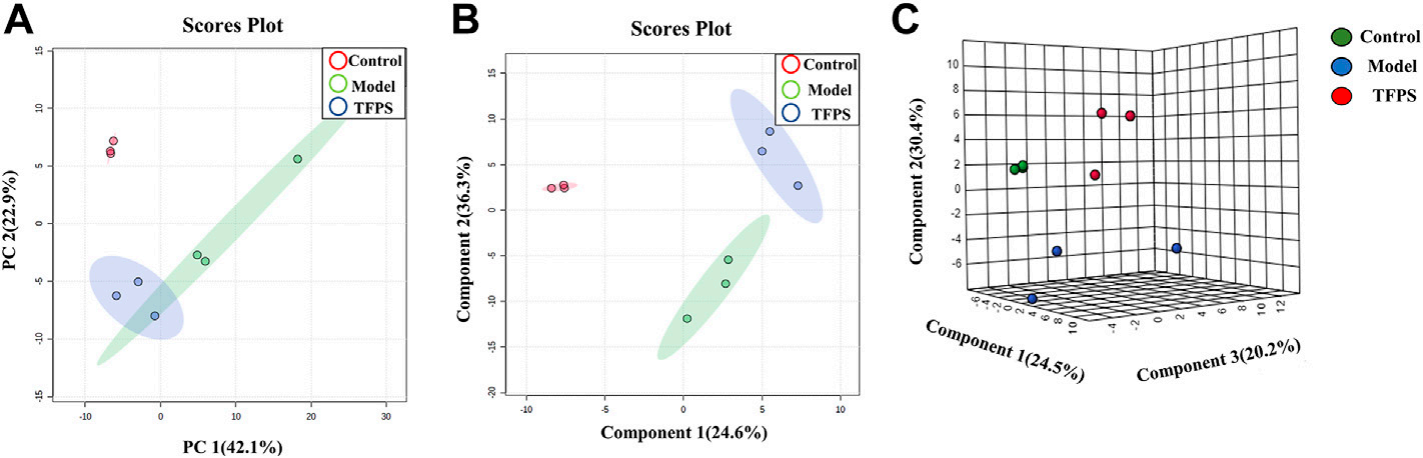
PCA and partial least squares discriminant analysis (PLS-DA). **(A)** The PCA scores plot of PC1 (first principal component) vs. PC2 (second principal component) showing the separation between control group (red), model group (green) and TFPS group (blue); **(B)** The PLS-DA score plot of control group (red), model group (green) and TFPS group (blue); **(C)** PLS-DA (3D) spots of control group (green), model group (blue) and TFPS group (red).

Having established that oral administration of TFPS is superior to topical application for suppressing the development of AD, we further investigated whether the therapeutic effect of oral TFPS on DNFB-induced AD is associated with the regulation of metabolism in mice. Animal feces were analyzed for metabolites by using the principal component analysis (PCA), which is an unsupervised pattern recognition method used to reduce the dimensionality of UPLC-MS data and reveal the inherent clustering of samples. Additionally, the PCA was used to summarize the feces metabolic phenotypes and compare composition of metabolites between the control group, model group, and TFPS group. The PCA score chart shows that the metabolite clusters of the control group and the model group are evidently separated, and the trend of separation between the TFPS group and the model group is also obvious (Figure 8A). In addition, the partial least squares-discriminant analysis (PLS-DA) was used to perform classification and regression on the high-dimensional feces metabolomics data.

As shown in Figures 8B–C, the PLS-DA score shows an evident separation among the control, model and TFPS treatment groups, indicating that DNFB irritation has induced significant changes in animal feces metabolites, and TFPS therapy restored the normal metabolite composition in mice feces.

### Screening and identification of potential biomarkers

**FIGURE 9:**
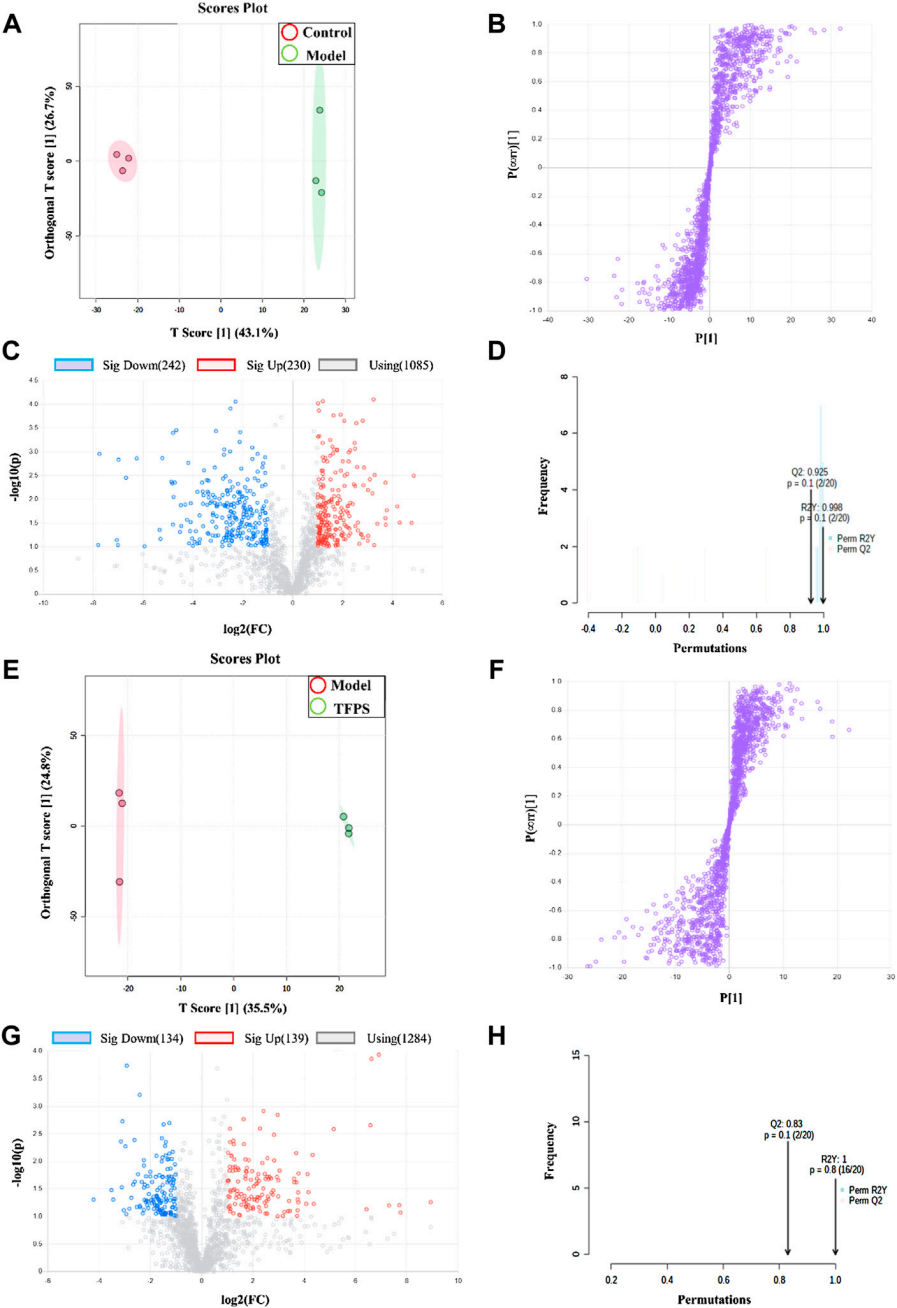
OPLS-DA analysis on experimental groups. **(A)** OPLS-DA score chart for control and model groups. **(B)** Statistically verified scatter plot was obtained through 2000X replacement test for control and model groups. **(C)** OPLS-DA S-plot of metabolic profiling for control and model groups. **(D)** Volcano chart by the OPLS-DA analysis for control and model groups. **(E)** OPLS-DA score plot for model and TFPS groups. **(F)** Statistically verified scatter plot was obtained by 2000X permutation test for model and TFPS groups. **(G)** OPLS-DA S-plot of metabolic profiling for model and TFPS groups. **(H)** Volcano plot for model and TFPS groups.

The multivariate analysis was carried out to explore specific differential metabolites. Differential metabolites were successfully identified among the normal control, modelling and TFPS treatment groups by using the OPLS-DA software package. The obtained scores showed complete separation of metabolic profiles between the control and model groups (Figure 9A), or between the model and TFPS treatment groups (Figure 9E), indicating the existence of potential metabolic biomarkers for DNFB-induced AD-like condition. The OPLS-DA analysis also obtained a good model explanation and prediction by the 2000X permutation test, with *R* ^2^ = 0.998 and *Q* ^2^ = 0.925 for control and model groups, and *R* ^2^ = 1, *Q* ^2^ = 0.83 for model and TFPS groups (Figures 8B,F).

**FIGURE 10:**
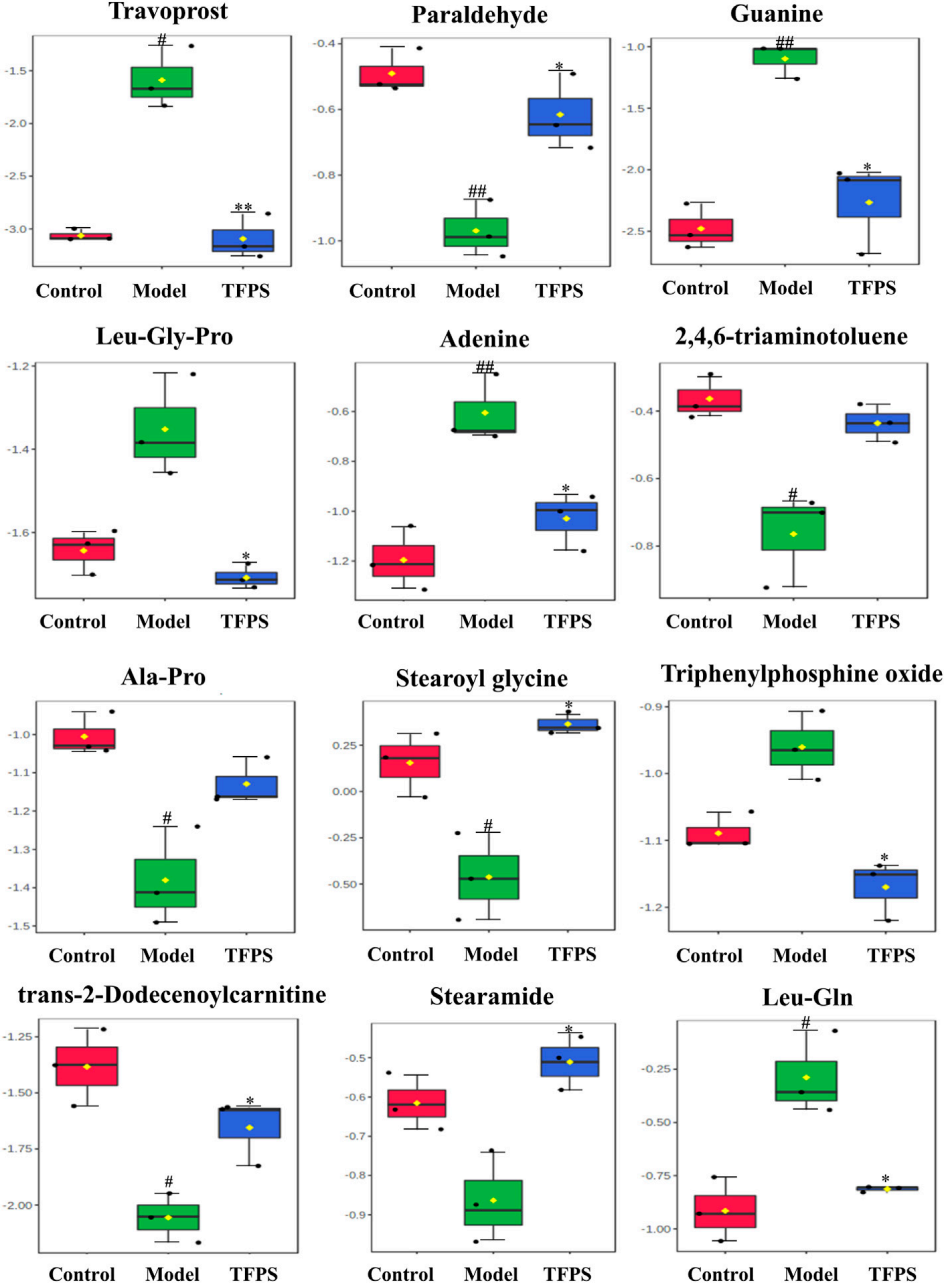
Changes of potential metabolite biomarkers by TFPS treatment in DNFB-induced AD-like mice. All values are presented as means ± S.E.M (*n* = 3). ^#^ *p* < 0.05, ^##^ *p* < 0.01 compared with the control group; ∗*p* < 0.05, ∗∗*p* < 0.01compared with the model group.

In S-plots and volcano curves (Figures 9C,D,G,H), differential metabolites were visualized and filtered through the OPLS-DA mode, and 12 potential biomarkers that were significantly regulated by high-dose of oral TFPS were screened and identified (Table 2; Figure 10). The TFPS modulation biomarkers from the feces include travoprost, paraldehyde, guanine, Leu-Gly-Pro, adenine, 2,4,6-triaminotoluene, Ala-Pro, stearoylglycine, triphenylphosphine oxide, trans-2-dodecenoylcarnitine, stearamide and Leu-Gln.

**TABLE 2:** Identified potential biomarkers regulated by TFPS treatment.

	RT [min]	Calc. MW	Formula	HMDB ID
Travoprost	7.644	500.23775	C26 H35 F3 O6	0014432
Paraldehyde	0.984	132.07892	C6 H12 O3	0032456
Guanine	1.990	151.04915	C5 H5 N5 O	0000132
Leu-Gly-Pro	8.474	285.16825	C13 H23 N3 O4	
Adenine	2.690	135.05435	C5 H5 N5	0000034
2,4,6-triaminotoluene	0.757	137.09516	C7 H11 N3	0247627
Ala-Pro	6.645	186.10013	C8 H14 N2 O3	
Stearoylglycine	13.205	341.2922	C20 H39 N O3	0013308
Triphenylphosphine oxide	11.726	278.08545	C18H15OP	0259265
trans-2-Dodecenoylcarnitine	12.930	341.25597	C19 H35 N O4	0013326
Stearamide	13.371	283.28686	C18 H37 N O	0034146
Leu-Gln	6.844	259.15267	C11 H21 N3 O4	0028927

### Changes in short-chain fatty acidss after *Tremella fuciformis* polysaccharides-treatment

**FIGURE 11:**
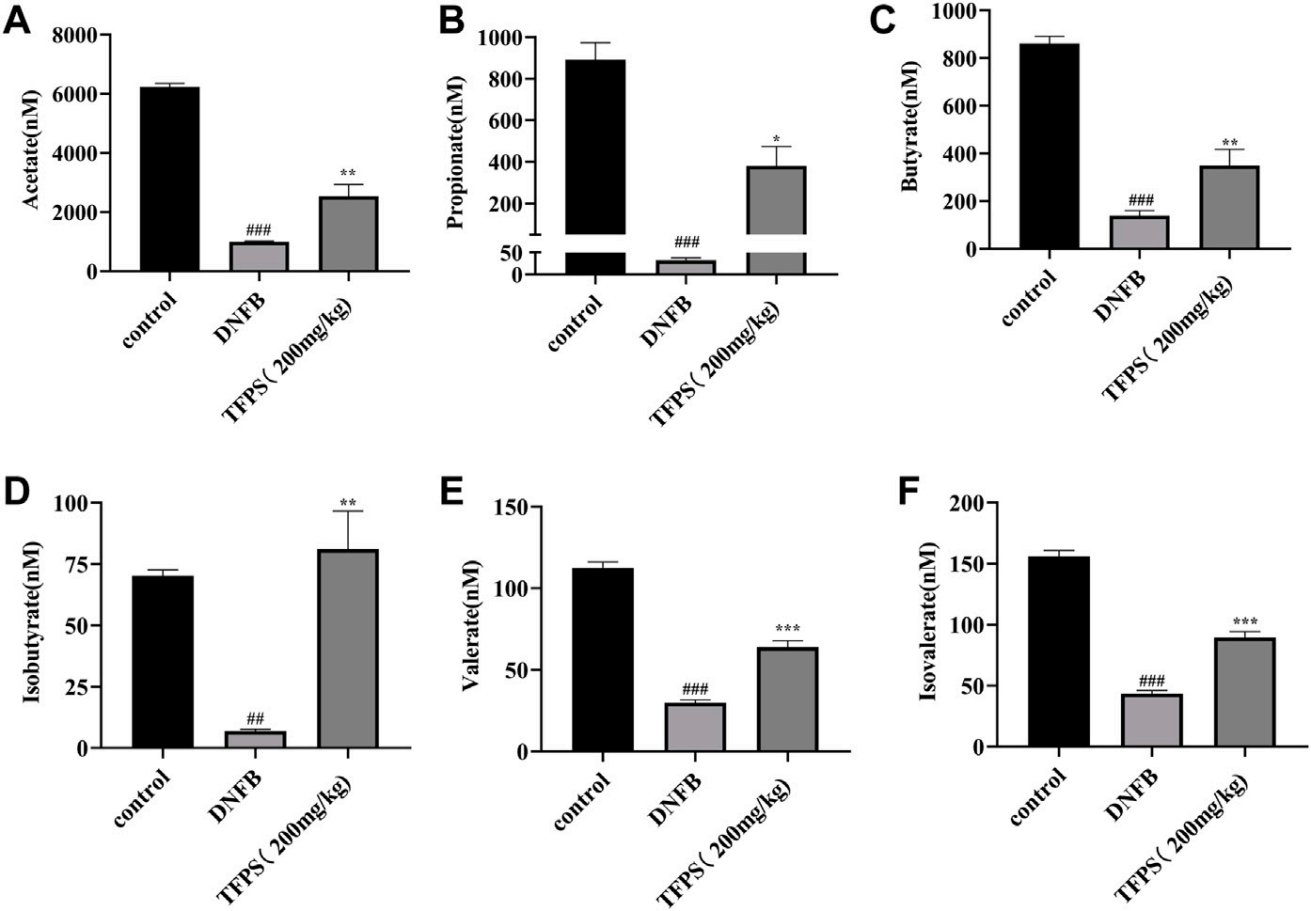
Changes of SCFAs by TFPS treatment in DNFB-induced AD mice. **(A)** Measurement of concentrations of acetate **(A)**, propionate **(B)**, butyrate **(C)**, isobutyrate **(D)**, valerate **(E)** and isovalerate **(F)** in control-, DNFB induction- and TFPS treatment groups, respectively. All values are presented as means ± S.E.M (*n* = 3). ^##^ *p* < 0.01, ^###^ *p* < 0.001 compared with the control group; ∗*p* < 0.05, ∗∗*p* < 0.01, ∗∗∗*p* < 0.01compared with the model group.

Gut microbiota-derived metabolites, including short-chain fatty acids (SCFAs), are involved in maintaining epithelial barrier homeostasis and modulating inflammatory responses. As shown in Figure 11, the concentrations of acetate, propionate, butyrate, isobutyrate, valerate and isovalerate were all significantly decreased in DNFB-induced AD mice comparing with the control group (Figures 11A–F). However, the TFPS treatment restored the reduced levels of acetate, propionate, butyrate, isobutyrate, valerate and isovalerate in DNFB-induced AD groups. These findings suggest that the modulation of gut microbial-derived SCFAs by TFPS may contribute to the alleviation of AD.

### Correlation between Atopic dermatitis symptoms, relative abundance of gut microbiota and metabolite changes in Atopic dermatitis mice

**FIGURE 12:**
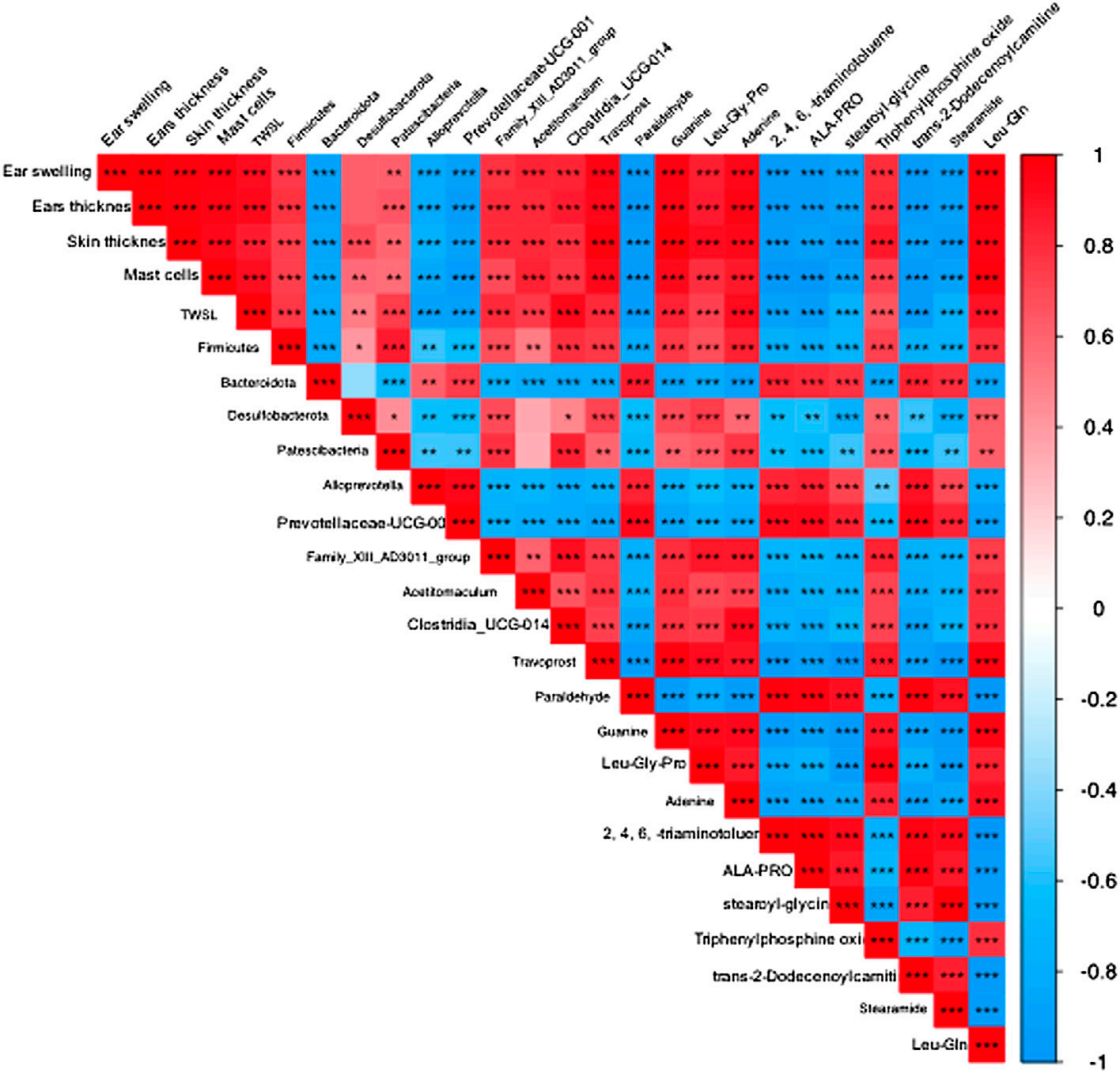
Correlation among AD symptoms, relative abundance of gut bacterial and metabolite changes. The correlation was determined using the Spearman’s rank correlation analysis. The red color indicates positive correlation, and the blue color indicates negative correlation. ∗*p* < 0.05, ∗∗*p* < 0.01, ∗∗∗*p* < 0.001.

To explain the relationship between AD severity, relative abundance of gut microbiota, and metabolite alterations, we performed the Spearman rank correlation analysis on AD-related conditions like ear swelling, ear thickness, skin thickness, mast cell counting, TEWL, expression levels of TNF-α, IFN-γ and IgE, relative abundance of gut microbiota, metabolites and SCFAs (Figure 12). At the genus level, the abundance of *Prevotellaceae-UCG-001* that belongs to the phylum *Bacteroides* is significantly and negatively correlated with the ear swelling, ear thickness, skin thickness, mast cell counting, TEWL, TNF-α and IFN-γ expressions (*p* < 0.05). It was also observed that the abundance of *Family XⅢ AD3011_group* is significantly and positively correlated with the AD-like conditions including ear thickness, IgE level (*p* < 0.05) and IFN-γ secretion (*p* < 0.01). Similarly, the abundance of *Clostridia_UCG-014* is significantly and positively correlated with ear swelling, ear thickness, TEWL, expression levels of TNF-α (*p* < 0.05) and IFN-γ (*p* < 0.001). Moreover, at the genus level, the beneficial bacteria *Prevotellaceae-UCG-001* were significantly and positively correlated with metabolites paraldehyde, 2,4,6-triaminotoluene, Ala-Pro, stearoyl glycine (*p* < 0.05) and trans-2-dodecenoylcarnitine (*p* < 0.01), and negatively correlated with travoprost and Leu-Gln (*p* < 0.05). Additionally, SCFAs were found to be significantly and negatively correlated with the severity of AD symptoms, levels of TNF-α, IFN-γ and IgE (*p* < 0.01), while the abundance of prevotellaceae-UCG-001 appeared positively correlated with SCFA levels. The above results suggest that the TFPS treatment is beneficial to ameliorate DNFB-induced AD-like symptoms through modulation of gut microbiota in mice.

## Discussion

AD is a common chronic inflammatory skin disease characterized by an impaired Th2 immune response. In recent years, the associations between gut flora disturbances and atopic dermatitis have been identified (Kukkonen et al., 2007; Behera et al., 2020). Our study demonstrated that oral administration of TFPS was able to alleviate the pathological severity of DNFB-induced AD-like condition in mice. The underlying mechanisms appear to be related to the increase of Treg cells and the regulation of intestinal microbiota and their metabolites.

In this study, we examined AD treatment by topical application or oral administration of TFPS. Previously, Essendoubi et al., revealed that the hyaluronic acid (HA) with 20–300 kDa of low molecular weights could pass through the stratum corneum, by contrast, the higher molecular weight of HA fractions (1,000–1,400 kDa) showed the evident impermeability of skin (Essendoubi et al., 2016). The TFPS that we have extracted range from 30 to 1,230 kDa in sizes, partially distributing in the range of 20–300kDa, so at least partially and potentially penetrate the skin and function to alleviate DNFB-induced AD-like conditions. And this may provide one reason for explaining the observed improvement of AD condition through topical application of the TFPS preparations in this study.

We have to point out that the previous HA penetration was examined on normal skin. And in our study, the TFPS was applied on DNFB irritation damaged skin, which may allow the higher molecular weight of TFPS (>300 kDa) to penetrate the skin epithelium. Additionally, it is possible that the topically applied larger fraction of TFPS (>300 kDa) may constitute a moisturizing polysaccharide coating/hydrogel on the damaged animal skin, which possibly improves the re-epithelialization of AD skin.

Blocking receptors for IL-4 and IL-13 with dupilumab has been described to lead to improvement of AD well before IgE levels change.

The pathogenesis of AD is involved in both genetic and environmental causes (Kwon et al., 2018). Th2 cells may be triggered to secrete excessive interleukin (IL) 4 and IL-13 and increase specific IgE production by polycyclic aromatic hydrocarbons in polluted air, which induced mast cells and basophils to release inflammatory factors leading to the onset of AD (Werfel et al., 2016). Meanwhile, IFN-γ and other pro-inflammatory cytokines, such as TNF-α, are abundant in the chronic phase of AD (Suárez-Fariñas et al., 2013). TNF-α and IFN-γ are able to induce an increase in pro-inflammatory mediators such as IL-6 and COX-2 in HaCaT cells (Lee et al., 2020). Therefore, the inhibitory effects of TFPS on the production of proinflammatory cytokines can be one of the mechanisms by which TFPS treatment attenuate the development of AD symptoms such as epidermal thickening and mast cell infiltration.

Increasing evidences have demonstrated that the development and progression of AD are closely correlated to the dysregulation in the composition and diversity of intestinal flora (Stokholm et al., 2018; Fang et al., 2021; Lopez-Santamarina et al., 2021). Metabolites from intestinal microbiota may stimulate activation of immune cells in host, such as short-chain fatty acid (SCFA), vitamins and amino acids (O’Hara and Shanahan, 2006). This observation indicates that the gut flora can be targeted to modulate host immunity to alleviate the clinical symptoms of AD. Actually, the correlation of AD with gut microbiota was well documented via sequencing analysis and the “gut-skin” axis has been recognized as an important target for developing novel AD therapies.

The diversity of intestinal flora is decreased in patients with AD compared to healthy populations. It was shown that the intestinal flora of AD patients had a significantly higher proportion of *Clostridia*, *Clostridium difficile*, *Escherichia coli*, and *Staphylococcus aureus (S. aureus)*, than healthy controls, while the proportion of *Bifidobacteria*, *Bacteroidetes*, and *Bacteroides* was reduced (Lee et al., 2018). Kalliomäki et al. demonstrated that *Lactobacillus* can effectively prevent early atopic dermatitis in high-risk children through modulation of intestinal flora by giving *Lactobacillus* GG to mothers who had at least one first-degree relative (or partner) with atopic eczema, allergic rhinitis, or asthma prenatally, and to their infants for 6 months postnatally (Kukkonen et al., 2007). Thus, modulating the diversity and composition of the intestinal flora may reduce the occurrence and progression of AD as an alternative approach to reduce adverse drug reactions of AD treatment.

A recent study has shown that high dose of *T. fuciformis* polysaccharides (HTPs) can alleviate dextran sodium sulfate (DSS)-induced colitis in mice through modulation of immune response and intestinal flora (Xu et al., 2021b). However, TFPS has never been examined for their effects on alleviation of AD through modulation of intestinal flora. Our study found that Oral administration of TFPS reduced the F/B ratio of intestinal flora in DNFB-induced mice compared to the model group. This suggests that the TFPS therapy could rescue the intestinal microbial dysbiosis induced by DNFB and bring it back to normal condition. To our best knowledge, this is the first study revealing TFPS therapeutic efficacy on AD via regulating the gut-skin axis.

The increase in *Firmicutes/Bacteroidetes* (F/B) ratio was also associated with the maintenance of normal intestinal homeostasis and consequently increased the concentration of SCFAs in the gut (Dei-Cas et al., 2020). SCFAs, as the most abundant microbial metabolites in the colonic lumen, are the main energy source for epithelial cells and affect the expression of genes required to maintain epithelial barrier and defense functions. They regulate the activity of innate immune cells such as macrophages, neutrophils and dendritic cells, and antigen-specific adaptive immunity mediated by T and B cells (Ellis et al., 2019). Among SCFAs, butyrate is the preferred fuel for the colonic epithelial cells, which stimulates the differentiation of regulatory T cells (Tregs) to maintain the balance between Th1 and Th2 (Tanoue et al., 2016).

*Alloprevotella*, *Prevotellaceae-UCG-001*, *Acetitomaculum*, *Family XIII AD3011_group* and *Clostridia_UCG-014* were the most affected microbial genera in the TFPS treatment group. *Alloprevotella* belonging to the phylum *Bacteroidetes* has shown anti-inflammatory effects (Ning et al., 2020). Moreover, Prevotellaceae-UCG-001 that belongs to the Prevotellaceae family, a Gram-negative anaerobic bacteria, produces SCFAs in the gut microbiota (Liu et al., 2021). Also, DNFB-induced AD mice showed reduction in the number of bacteria, which have anti-inflammatory and SCFAs-producing capacities, compared with the control animals. However, the TFPS treatment effectively regulated the DNFB disturbed intestinal microbiome, and recovered the composition of gut microbes almost back to normal level in control animals. These observations thus suggest that the gut microbiome and its derived metabolites like SCFAs, are essential for the maintenance of skin health. Of note, among the 12 identified potential biomarkers, the change in purine metabolism is most pronounced. Purines have important biosynthetic functions, such as the formation of nucleic acids, DNA and nucleic acid monomer precursors. Mycophenolic acid (MPA), an inhibitor of purine synthesis, has been clinically used to treat severe AD (Knol et al., 2012). Interestingly, our results indicated that the TFPS treatment significantly inhibited DNFB-induced purine elevation in AD-like mice. This may be a potential mechanism by which TFPS intervenes in AD. Overall, this study provides a potential strategy to use natural food products for the prevention and treatment of AD via modulation of gut microbial metabolism and immune responses.

This study demonstrated the potential of using oral TFPS for AD treatment. However, some issues need to be elucidated for the clinical application of TFPS. First, the animal model effectiveness cannot guarantee the similar therapeutic efficacy in human diseases, and thus next clinical trials must be performed before putting forward the product to medical practice. Second, the animal model dosage may not work for human AD treatment and the therapeutic safety has to be determined in human cases. Third, the oral TFPS formulation needs to be optimized. Fourth, the patient selection criteria must be established for the precision medicinal application of oral TFPS.

## Conclusion

*T. fuciformis* polysaccharides (TFPS) were successfully extracted by hot distilled water. Both topical application and oral administration of TFPS are effective for treating DNFB-induced AD-like conditions in mice, however the latter demonstrates significantly better efficacy. The AD-attenuation effects of TFPS are associated with the upregulation of Treg cell number and restoration of gut microbiota and metabolites, such as SCFAs. Moreover, 12 metabolites were found being significantly modulated by TFPS treatment and thus identified as potential AD biomarkers. These observations suggest that the oral administration of TFPS potentially constitutes a novel AD therapy.

## Data Availability

The original contributions presented in the study are included in the article/Supplementary Material, further inquiries can be directed to the corresponding authors.
