# Supplementary material for: Tremella fuciformis polysaccharides alleviate induced atopic dermatitis in mice by regulating immune response and gut microbiota
Source: Front Pharmacol. 2022 Aug 25;13:944801. doi: 10.3389/fphar.2022.944801 (PMC9452665; doi:10.3389/fphar.2022.944801)
Supplement: Supplementary file 1 [file Table1.DOCX]

|  | Control | Model | TFPS |
| --- | --- | --- | --- |
| Shannon | 2.872±0.090 | 2.602±0.018 | 2.533±0.053 |
| Simpson | 0.105±0.011 | 0.130±0.004 | 0.166±0.017 |
| Ace | 91.513±3.081 | 92.001±1.273 | 89.299±0.765 |
| Chao 1 | 88.900±2.456 | 89.433±1.402 | 87.621±1.218 |
