# Supplementary material for: Tremella fuciformis polysaccharides alleviate induced atopic dermatitis in mice by regulating immune response and gut microbiota
Source: Front Pharmacol. 2022 Aug 25;13:944801. doi: 10.3389/fphar.2022.944801 (PMC9452665; doi:10.3389/fphar.2022.944801)
Supplement: Supplementary file 2 [file Table2.DOCX]

|  | RT [min] | Calc. MW | Formula | HMDB  ID |
| --- | --- | --- | --- | --- |
| Travoprost | 7.644 | 500.23775 | C26 H35 F3 O6 | 0014432 |
| Paraldehyde | 0.984 | 132.07892 | C6 H12 O3 | 0032456 |
| Guanine | 1.990 | 151.04915 | C5 H5 N5 O | 0000132 |
| Leu-Gly-Pro | 8.474 | 285.16825 | C13 H23 N3 O4 |  |
| Adenine | 2.690 | 135.05435 | C5 H5 N5 | 0000034 |
| 2,4,6-triaminotoluene | 0.757 | 137.09516 | C7 H11 N3 | 0247627 |
| Ala-Pro | 6.645 | 186.10013 | C8 H14 N2 O3 |  |
| Stearoylglycine | 13.205 | 341.2922 | C20 H39 N O3 | 0013308 |
| Triphenylphosphine oxide | 11.726 | 278.08545 | C18H15OP | 0259265 |
| trans-2-Dodecenoylcarnitine | 12.930 | 341.25597 | C19 H35 N O4 | 0013326 |
| Stearamide | 13.371 | 283.28686 | C18 H37 N O | 0034146 |
| Leu-Gln | 6.844 | 259.15267 | C11 H21 N3 O4 | 0028927 |
